# Melanotic Sarcoma of the Choroid

**Published:** 1874-03

**Authors:** F. C. Hotz


### MELANOTIC SARCOMA OF THE CHOROID.

CLINICAL LECTURE, OPHTHALMIC WARD COOK COUNTY HOSPITAL. SERVICE PROF. F. C. HOTZ, JANUARY 14, 1874.

*Reported by D. A. K. Steele, M.D., House Surgeon.*

GENTLEMEN: To-day I take pleasure in demonstrating to you two tumors, greatly differing from each other in their position and in their bearing upon the eye-ball. Three weeks ago this young man, twenty-six years of age, had the lower lid of his left eye burned by a piece of red-hot iron, which flew up and fell upon the conjunctival surface of the lid. The inflammation following the injury passed away in one week; but a small knob was then observed to grow out of the lower lid. Within two weeks this knob, as you see, has attained the size of a large pea. It is smooth, pretty solid, and attached to the lid by a very small pedicle, similar to the nasal and aural polypi. You will also notice that the conjunctiva is tightly drawn towards the root of the polypoid tumor by radiating cicatrices.

This observation will at once explain to us the development of the conjunctival polypus. The conjunctiva had been extensively scorched; and when the wound was healing over, with expithelium from the edges, the granulations grew luxuriantly in the center, and soon rose above the level of the conjunctiva. By the subsequent contraction of the cicatrix, the bases of these granulations were constricted to a thin pedicle. But they continued to grow larger and firmer; and if we examine the polypus under the microscope, we shall see the structure of young connective tissue just beginning to be consolidated. This tumor, gentlemen, although it has grown very rapidly, is of a very innocent nature; and its removal is indicated by the annoyance and unsightliness, rather than by any malignancy. It acts like a foreign substance, causing a slight irritation of the eye, as you can notice by the redness of the conjunctiva, and the somewhat increased secretion of tears; but it would never endanger the functions of the eye—it would never impair vision. The removal of this tumor is the easiest thing in the world. While an assistant is everting the lower lid, I shall grasp the body of the growth with a forceps, and snip the pedicle, closely to the conjunctiva, with a pair of scissors. This is the simplest, safest, and surest treatment; and I would not indorse the treatment of that physician who, as the patient told me, applied nitrate of silver on the tumor; because it could not be prevented from spreading over the conjunctiva, causing an unnecessary irritation of that mucous membrane; but especially because nitrate of silver is known to favor the rapid growth of polypi, rather than to destroy them.

[After the operation was done, and the little tumor passed around, Dr. H. continued]:

And now, gentlemen, look at this old gentleman, who, in his fifty-second year of age, has come to us, from a great distance, hoping to find relief from his sufferings. Ten years ago he lost the sight of his eye, within a short time, although there was no pain, nor any sign of inflammation. The blind eye looked just like the other, and continued so until, four years ago, the lens became cataractous. About this time he began to have intermittent pain in the blind eye, most severe at night. One year ago the eye began to grow larger, and the pain became more intense, extending over the left side of the face and head. Patient had always enjoyed good health; and, in spite of the many restless nights he had, his appearance is good for his age. Now, let us examine this eye: the upper lid is greatly swollen, and red, but soft; raising this œdematous lid, we observe a transparent, but abnormally small, cornea, and a discolored iris, which is crowded up to the posterior surface of the cornea, so that no anterior chamber exists; the pupil is converted into a small, irregular gray dot. The most striking feature, however, is the change of the shape of the eye-ball. It is a ball no longer: it is an irregular, knotted body. At a short distance from the cornea, three protuberances rise under the ocular conjunctiva, which is blended with their highest part. One of them occupies the upper inner section of the sclerotic; one the upper external, and the third the lower external portion. The two upper are perfectly black; the lower one has a light, grayish color; all three are solid, and pass over into the globe gradually and imperceptibly. The eye-ball itself is also hard, like a solid mass. Its rotations are very defective, as it cannot be turned outward nor upward either. The conjunctiva is highly congested. The diagnosis of the case certainly is very easy; and we need not hesitate to pronounce it a case of *melanotic sarcoma*; melanotic (dark-colored), because of the abundance of brown and black pigment; sarcoma, because in the eyes of adults this kind of morbid growth is found most frequently—nay, almost exclusively. These tumors originate in the choroid, and their presence is unknown to the patient for some time (latent stage of the tumor). As they grow larger, they form a rounded tumor, which projects into the vitreous humor, and carries the retina with it. This then gives rise to an irritation of the retina, causing a dimness of sight, and finally leads to a complete detachment of the retina, with total loss of the sight.

This happened to our patient ten years ago; and, therefore, the tumor is older than ten years. For so long a time, it has grown very slowly indeed. It took over six years to fill up the whole interior of the globe; for, during this period, the eye appeared like the right one. But, as soon as the bulk of the tumor becomes larger than the cavity of the eye-ball, it begins to press against the sclerotic, trying to expand it. At this time, the eye becomes hard, inflamed; and the patient is tormented by violent ciliary neurosis. Four years back, the eye of the patient passed into this stage. This period lasted three years. At last, the shell of the eyeball gives way to the pressure of the morbid growth at some point; either because the cornea sloughs, or because the sclerotic is softened and perforated. A protuberance then appears under the conjunctiva—first noticed by our patient one year ago. From this time the neoplasma generally grows more rapidly; and soon, perforating the conjunctiva, exhibits an ulcerated, nasty surface, in the meanwhile, also, extending deeper into the orbit. Our tumor has not yet broken through the conjunctiva, but it is very nearly ready to do so; and the considerable disturbance of the mobility of the eye indicates a disagreeable extension of the tumor into the cellular tissue and muscles of the orbit.

Reviewing the history of this case, you find that the first period has lasted over six years; the second stage three years; the third only one year. Thus, it confirms the observations of others, that the first period always is the longest. Although the sarcoma does not influence the health of the patient, during the three periods mentioned of its growth, it is not so harmless, after all. It remains a local disease for many years, especially as long as it is inclosed in the eye-ball; but, gone beyond this inclosure, it sooner or later sends its germs to distant vital organs; and the fourth act of the drama, with which it concludes, is the death of the patient, caused by the development of sarcomatous tumors in the liver, lungs, pleura, kidneys, stomach, or in the brain. I am sorry to say, that I do not expect that our patient will escape his fate. Still, I propose to operate on him, for these reasons: the sarcomatous tumors in the organs are started by germs, carried there from the orbit by the lymph and blood. By the removal of the maternal tumor, we stop the source of infection, either permanently, if we can remove every particle of morbid growth, or, at least, for some time, if particles are left in the orbit which give rise to a recidive tumor. In this case, we may prolong the life of the patient; but suppose his internal organs have, by this time, been infected (although we cannot discover the beginning of this calamity); then, of course, the operation could not delay the death by a single day; and still, I would perform it, to make the last days of his life more comfortable, by stopping that fearful pain, which makes life intolerable to him. The removal of this eye-ball will not be a clean enucliation, because the ocular conjunctiva must be removed, and, most likely, a portion of the muscles, and the cellular tissue of the orbit, too. When the patient is well etherized, I shall first enlarge the field of the operation by splitting the external canthus; then I shall sever the ocular conjunctiva from the tarsal portions, by incisions through the retrotarsal folds; and then I shall dig my way into the orbit, to get to the posterior termination of the tumor. Having it loosened from the sound vicinity, I shall draw it out, in order to stretch the optic nerve as much as possible. The points of the scissors are then passed up to the optic foramen, where the optic nerve will be snipped through. The tumor taken out, the bleeding is arrested by pressing a piece of sponge into the orbital cavity. This sponge will be kept in for about an hour. The further treatment, after the operation, consists, mainly, in syringing the cavity with warm water.

*Anatomical Examination of the Eyeball.*—The eye, with adherent tumors, forms an irregular knobby body, somewhat oval in form; horizontal; diameter, one and three-fourths inches; antero-posterior, one and one-fourth inches; vertical, one inch. There are two tumors on external, and one on internal side, protruding through the sclerotic, and firmly adherent to conjunctiva, almost black in color. A similar knob-like mulberry protrudes from the posterior part of the eye, on the outer side of optic nerve. The optic nerve, for five-eighths of an inch long, is totally degenerated to a black substance similar to tumor. The eye was divided, by a meridianal section, from before backward. The sclerotic can be traced, on the external side, back a short distance from the cornea, and forward from the optic nerve. At the equator, it is dissolved in a grayish and encephaloid substance. Of the inner half of sclerotic, scarcely any trace is left. Its interior is completely filled with one uniform, black, soft mass, from the surface of which an inky fluid exudes, showing, under the microscope, a granular detritus of pigmented cells. The rotten tissue of iris, which was crowded to the cornea, cannot be separated from the black substance behind it. Where the original posterior pole of the lens was, a calcareous substance is imbedded in the tissue, evidently the petrified lens. The external lower part of tumor is less pigmented.

The eye and adherent tumors were hardened in a solution of chromic acid; a number of sections were made, and submitted to Professor L. Curtis for microscopical examination. The tumors were pronounced to be melanotic, round, or granulation-celled sarcoma, in structure.

[Two weeks later (January 28th), while the tumor was passed around, and its microscopical anatomy demonstrated, Dr. H. remarked]:

The patient has nicely recovered from the operation, and left for his home yesterday. The whole wound is covered by red, sound granulations, secreting a moderate quantity of thick, creamy pus. Four or five days after the operation, however, the granulations had not so satisfactory an appearance. Throughout, from the bottom of the cavity, as well as from under the upper lid, the granulations were growing out, in rounded, solid protuberances, of a grayish red color. There we had the immediate development of sarcomatous lumps, by germs left in the orbital tissues. I then resorted to a remedy that, within the last year, had pleased me very much, in similar cases, by destroying the germs of malignant growths, and thereby preventing the return of the neoplasma: *in loco.*, with a camel's hair brush, the solution of

℞.—Acid carbolic, ʒ j.

Alcohol, ʒ ij.

Aq. dist., ℥ ss.

Tinct. iodine, ʒ ss.

was applied, daily, to the suspicious looking granulations; and under this treatment they gradually disappeared, and in their place healthy granulations covered the ground, which, I hope, will consolidate in a sound catrization.

